# Baru Almonds Increase the Activity of Glutathione Peroxidase in Overweight and Obese Women: A Randomized, Placebo-Controlled Trial

**DOI:** 10.3390/nu11081750

**Published:** 2019-07-30

**Authors:** Rávila Graziany Machado de Souza, Aline Corado Gomes, Anderson Marliere Navarro, Luiz Carlos da Cunha, Marina Alves Coelho Silva, Fernando Barbosa Junior, João Felipe Mota

**Affiliations:** 1 Clinical and Sports Nutrition Research Laboratory (LABINCE), Faculty of Nutrition, Federal University of Goias—UFG, Goiânia, 74605-080 GO, Brazil; 2 Department of Health Sciences; Faculty of Medicine, University of São Paulo—USP, Ribeirão Preto, 14049-900 SP, Brazil; 3 Nucleus of Toxic-pharmacological Studies and Research (NEPET), Federal University of Goiás—UFG, Goiânia, 74605-220 GO, Brazil; 4 Faculty of Pharmaceutical Sciences of Ribeirão Preto, University of São Paulo—FCRP/USP, Ribeirão Preto, 14040-903 SP, Brazil

**Keywords:** nuts, oxidative stress, overweight, inflammation, minerals

## Abstract

Background: Obesity-induced inflammation is frequently associated with higher oxidative stress. In vitro and experimental studies have considered baru almonds (Dipteryx alata Vog) as a legume seed with high antioxidant capacity. The aim of this study was to evaluate whether baru almonds are capable of improving the inflammatory and antioxidant status in overweight and obese women. Methods: In a parallel-arm, randomized placebo-controlled trial, 46 overweight and obese women (age: 40 ± 11 years; body mass index: 33.3 ± 4.3) were randomly assigned to receive advice to follow a normocaloric and isoenergetic diet with placebo (PLA, *n* = 22) or similar advice plus 20 g baru almonds (BARU, *n* = 24) for 8 wk. Malondialdehyde (MDA), adiponectin, tumor necrosis factor-α, interleukin-6, interleukin-10, antioxidant enzymes activities (catalase—CAT; glutathione peroxidase—GPx; superoxide dismutase—SOD), and minerals were analyzed in plasma samples. Results: At baseline, groups were similar regarding the body composition, oxidative, and inflammatory parameters. The BARU group increased the activity of GPx (+0.08 U/mg, 95%CI + 0.05 to +0.12 vs. −0.07, 95%CI −0.12 to −0.03, *p* < 0.01) and plasma copper concentration (*p* = 0.037) when compared to the PLA group. No differences were observed between groups in CAT and SOD activity or MDA and cytokines concentrations. Conclusions: Baru almond supplementation increased the GPx activity in overweight and obese women.

## 1. Introduction

Overweight and obesity are associated with elevated secretion of pro-inflammatory cytokines and reactive oxygen species [1]. Oxidative stress is an imbalance between free radical production and antioxidants, which increases the risk of cardiovascular diseases [2,3]. Reactive oxygen species cause the overexpression of nicotinamide adenine dinucleotide phosphate (NADPH) oxidase and the reduced activity of antioxidant enzymes, which releases pro-inflammatory adipocytokine [4]. Moreover, high concentrations of pro-inflammatory cytokines, such as interleukin-6 (IL-6) and tumor necrosis factor-α (TNF-α), induce a positive reciprocal feedback loop in oxidative stress thereby increasing reactive oxygen species production [5].

The consumption of tree nuts, walnuts, and legume seeds is associated with a reduction in oxidative stress and inflammation [6]. This effect is associated with the presence of bioactive compounds, such as phenolic compounds, carotenoids, tocopherols, phospholipids, dietary fibers, monounsaturated and polyunsaturated fatty acids (MUFA and PUFA, respectively), and some minerals (e.g., selenium, zinc, and copper) [7,8,9,10,11,12].

Baru tree (*Dipteryx alata Vog*.), a native plant species of the Brazilian cerrado, produces an edible dark brown seed, named “baru almonds” [13]. The growing trend of scientific interest in baru almonds is due to its nutritional composition [12]. Baru almond is a legume seed that contain high amounts of MUFA (51%), PUFA (31%), and protein (30%); and low amounts of saturated fatty acids (SFA—12%) and carbohydrate (12%). Baru almonds also present 12.5% of dietary fiber and high levels of calcium, iron, phytates, tannins, vitamin E, and zinc [12,14].

In an experimental study carried out with rats, the treatment with baru almonds as 10% of the diet resulted in a reduction in the oxidative stress [13]. In our previous clinical trial, we showed that diet supplementation with a baru almonds improved HDL and reduced abdominal adiposity in overweight and obese women [15]. However, the effects of baru almonds on oxidative stress and pro-inflammatory cytokines were not evaluated. Thus, the aim of this study was to evaluate whether baru almonds are capable of improving the inflammatory and antioxidant status in overweight and obese women.

## 2. Materials and Methods

### 2.1. Design

This study is a part of the BARU study—a randomized, controlled parallel-group design performed in eight weeks. Subjects were randomly assigned (1:1) to compose either one of the following groups: PLA (placebo) or BARU (baru group). Group allocation was not discussed with the participants; however, it was likely clear to them which group they were assigned to, based on the intervention provided.

### 2.2. Subjects

Sixty overweight or obese women were recruited to take part in this study; however, 46 women concluded all assessments and finished the intervention. The inclusion criteria were nonsmokers and nonalcoholics who had BMIs (in kg/m^2^) ranging from 24.9 to 40 and were not taking any dietary supplements.

Potential subjects were not included in the study if they (1) were on weight loss diet/medication; (2) were diagnosed with any acute or chronic disease; (3) were taking medication that could affect the immune system; (4) were pregnant or planned to become pregnant; (5) had gastrointestinal surgery, hormone replacement therapy, or antibiotic treatment.

The study was approved by the Local Ethical Committee (protocol 044/2012), registered at the Brazilian Clinical Trials Registry (RBR—2wpryx), and all of the subjects signed the informed consent. All of the procedures were in accordance with the Helsinki Declaration revised in 2008.

### 2.3. Dietary Intervention and Compliance

Both groups received a normocaloric and isoenergetic diet prescription (25–30 kcal/kg). The macronutrient composition of diets was 50%–60% of carbohydrate, 20%–30% of fatty acids, and 15%–20% of protein. Registered dietitians provided individualized dietary advice according to the guidelines for healthy eating [16]. The diet plan consisted of six meals with the amount of each food, cooking techniques and a food substitution list. The energy content of the diet was individually calculated to match each participant’s estimated energy needs and adjusted for levels of physical activity [17]. Physical activity was assessed by the validated International Physical Activity Questionnaire.

The PLA group received 800 mg/day of maltodextrin dispensed in sachet, and the BARU group received 20 g (approximately 15 units of almonds) of roasted baru almonds. To reduce bias, we emphasized that baru almonds were being offered in two different forms: Traditional and powder. The supplements were delivered in closed packages. The nutritional composition of baru almond was previously described [6], and the placebo was chosen for not influencing the oxidative state and representing an increase of only 3.2 kcal/day in daily energy intake [18]. The amount of baru almonds was established according to the portions of seeds and nuts [19], which represents the average intake for this almond [20]. The baru almonds were roasted in a conventional electric oven [14] to inactivate any anti-nutritional factors present [18], vacuum packed, and stored without exposure to light.

Daily supplement compliance and dietary intake were monitored as follow: (1) weekly via telephone calls and monthly during routine consultations; (2) counting the remained empty packages when the participants returned to their consultation; (3) self-reported dietary intake by 24-h dietary recall conducted at the face-to-face interview by trained dietitians; (4) 3-day food record, including a weekend day; and (5) the increment in plasma oleic fatty acid after intervention. Dietary composition was analyzed using the AVANUTRI software.

### 2.4. Blood Sampling and Laboratory Methods

Blood samples were drawn from the antecubital vein in the arm after a 12-h fast. Samples were centrifuged at 3500 rpm for 10 min at 4 °C (Combate, C.E.L.M, São Paulo, Brazil) and stored at −80 °C until analysis. Alanine aminotransferase, aspartate aminotransferase, creatinine, γ-glutamyl transferase, urea, and uric acid were determined by automated enzymatic methods (VITROS 950 Xrl Chemistry System, Johnson & Johnson, NJ, USA).

Plasma cytokines (IL-6, IL-10, TNF-α, and adiponectin) were measured in duplicate using Luminex technology with Milliplex MAP (Merck Millipore, Darmstadt, Germany) multiplex magnetic bead-based antibody detection kits according to the manufacturer’s instructions. The intra-assay CV ranged from 1.6 to 4.0% and the inter-assay CV 10.0 to 18.3% for all cytokines measured. The antioxidant enzymes activities—catalase (CAT), glutathione peroxidase (GPX), and superoxide dismutase (SOD) were assessed in erythrocyte lysates using a microplate reader (BioTek Synergy HT, Winooski, VT, USA) [21]. Serum malondialdehyde (MDA) was measured by high performance liquid chromatography (HPLC) using a method previously described [22] with minor modifications.

Serum concentration of trace elements was determined by inductively coupled plasma mass spectrometry (ICP-MS), equipped with a reaction cell (DRC-ICP model ELAN DRC II, PERKIN ELMER Sciex, Norwalk, CT, USA). Samples were diluted (1:50) in a solution containing 0.01% (*v*/*v*) Triton X-100 and 0.05% (*v*/*v*) nitric acid. Rhodium (10 mg/L) was used as an internal standard. Calibration standards ranged from 0 to 50 g/L.

### 2.5. Statistical Analysis

The sample size was estimated using the G* Power software (version 3.0.10). Based on an increase in plasma GPx activity (effect size, d = 0.90) after the consumption of Brazil nut [23], a sample size of 21 participants would be needed in each group to give 80% power to detect a significant difference between BARU and placebo (with a two-sided type 1 error of 5%).

Data were expressed as mean ± standard deviation (SD). The Shapiro–Wilk test was performed to assess normality and Levene test was performed to assess homoscedasticity. Paired t-test was used to identify within-group differences. Differences between groups were assessed by repeated measures analysis of covariance (ANCOVA) with baseline values as a covariate and time as the repeated measure. The posthoc tests were performed with Sidak corrections. In case of non-normal distribution and/or non-homoscedastic data, log transformation was performed before further analysis. Macronutrients intake were adjusted for energy intake. The *p*-value < 0.05 was considered statistically significant. Statistical analyses were undertaken using SPSS v. 21.0 (IBM^®^, North Castle, CT, USA) and STATA v. 12 (StataCorp^®^, College Station, TX, USA).

## 3. Results

Eighty-six people were assessed according to the inclusion and exclusion criteria. After the criteria assessment, sixty subjects were randomized into placebo or BARU groups. However, forty-six completed the intervention and done all the assessments (PLA = 22 and BARU = 24) [15]. At the baseline, body composition was similar between groups (Table 1). The average age was 40 ± 11 and BMI ranged from 27 to 40 kg/m^2^. The addition of baru almonds increased intake of MUFA and PUFA and decreased the carbohydrate. However, dietary intake did not differ between groups (*p* > 0.05; Table 2). No side effects were reported by participants during the intervention period and the adherence to the supplementation was 100% in both groups. There were no changes in uric acid, γ-glutamyl transferase, alanine aminotransferase, aspartate aminotransferase, urea, and creatinine concentrations between groups (Table 3).

Glutathione peroxidase activity reduced in the PLA group (*p* < 0.01) and increased in the BARU group (*p* < 0.001) with difference between groups (PLA: −0.071 U/mg, 95%CI −0.116 to −0.027 vs. BARU: +0.083 U/mg, 95%CI +0.046 to +0.120; *p* < 0.01). The BARU group also showed an increased SOD activity (+1.41 U/mg, 95%CI +0.507 to +2.31, *p* = 0.02), but without difference between the PLA group (Table 3).

Throughout intervention, the BARU group had increased plasma copper concentrations compared to PLA (*p* = 0.03). Both groups had reduced plasma cobalt concentrations, but the PLA group had higher reduction when compared with the BARU group (*p* < 0.01). No difference was noted for zinc, calcium, iron, phosphorus, magnesium, manganese, selenium, and strontium (Table 4). No statistical changes were found between groups for MDA and cytokines concentrations.

## 4. Discussion

The present study showed that 20 g of baru almonds increased GPx activity and plasmatic copper levels. There is growing evidence that oxidative stress is related with obesity and its co-morbidities [1]. Antioxidant enzymes such as CAT, SOD, and GPx have an important role in reducing the oxidative stress and inhibition of inflammation associated with obesity [24]. Superoxide dismutation reactions (superoxide ion to hydrogen peroxide) catalyzed by SOD and the conversion of hydrogen peroxide to water by GPx and CAT are essential to prevent the cellular oxidative damage [4].

The presence of bioactive compounds in nuts and edible seeds may contribute to the reduction in oxidative stress and prevent cardiovascular damages [6,13]. An investigation observed a decrease in thiobarbituric acid reactive substances and an increase in the activity of GPx and SOD enzymes in rats fed with a hypercholesterolemic diet and pistachios for eight weeks [8]. The antioxidant activity of baru almonds was evaluated in vitro by the potential ferric ion reducing method and showed a positive correlation with the total polyphenol concentration, making it possible for increased capture of oxygen reactive species [13]. However, in mildly hypercholesterolemic patients, baru almonds did not show any effect on MDA concentration and SOD activity [20]. Possible explanations could be the uncontrolled physical activity habits between groups, that can increase the antioxidant status [25], and the adherence to the supplementation.

Our study showed that baru supplementation was able to increase GPx activity. An increase in GPx activity was also observed with a daily intake of one Brazil nut by patients on hemodialysis [9,26] and obese women [10]. Brazil nuts provide a rich natural source of selenium which increase selenium status and consequently GPx activity [10]. Baru almonds, despite having low concentrations of this mineral, were effective in increasing the activity of GPx. This fact can be justified by the higher concentration of phenolic compounds in its composition [11]. Baru almonds contain eight phenolic compounds: Ellagic acid, coumaric acid, caffeic acid, hydroxybenzoic acid, ferulic acid, gallic acid, catechin, and epicatechin; these are stable at the high roasting temperatures and may be involved in the effect on GPx activity [12]. In addition, in our previous study, we observed reduction of abdominal adiposity after intervention with baru almond [15], which may have contributed to the increase of GPx activity, since the reduction of body fat seems to reduce the downregulation in gene and protein expression from antioxidant enzymes such as GPx [27].

The consumption of baru almonds also increased plasmatic copper concentration in overweight women. Copper is a critical component for function and stability of SOD, an antioxidant enzyme that protects the body against the destructive effects of free radicals [28]. Low dietary copper increases the risk factors for colon cancer in healthy men [29]. Furthermore, a randomized crossover study with women showed that copper supplementation increased serum copper concentration and the activity of SOD. The supplementation also reduced the fibrinolytic factor plasminogen activator inhibitor type 1 [30]. Here, the BARU group increased the SOD activity; however, without difference between groups. However, despite the BARU group increased copper concentration, the concentration remained lower compared to other studies [31,32] and to the expected cut-off point [33], which might have contributed for the absence of effect on SOD activity. In general, obese individuals appear to have lower copper concentrations, probably due to the low intake of polyunsaturated fatty acids [34].

In this study, cobalt concentration decreased in both groups; however, the reduction was higher in the PLA group. There are no studies that have evaluated the relationship between nut consumption and serum cobalt concentration, but it is known that these foods are abundant in an inorganic form of cobalt, as happens with fish and green vegetables [35]. Cobalt is involved in catabolic detoxification process of hemoglobin by activating the expression of antioxidant enzymes, such as heme oxygenase-1 (HO-1), which stimulates the production of interleukin-10, an anti-inflammatory cytokine [35].

Here we do not observe changes in cytokines concentrations. A clinical study that evaluated subjects with metabolic syndrome also did not observe anti-inflammatory effect from the consumption of a mix of nuts [36]. Although evidence indicates that the consumption of nuts has an anti-inflammatory effect [37], probably a greater reduction of body fat is more related to the reduction of the inflammatory process than the consumption of these foods. A possible limitation of this study is that the serum bioactive compounds were not analyzed. In conclusion, this study reports that in a normocaloric diet, regular consumption of 20 g of baru almonds increased the activity of GPx and the concentration of copper in overweight and obese women. Future studies are needed to confirm the results and to investigate the effects of baru’s bioactive components on cardiovascular health.

## Figures and Tables

**Table 1:** Baseline characteristics of body composition.

	PLA (*n* = 22)	BARU (*n* = 24)	*P*
Body Mass Index (kg/m^2^)	33.3 ± 4.6	32.5 ± 4.3	0.73
Waist circumference (cm)	97.5 ± 10.4	94.6 ± 12.6	0.39
Fat mass (kg)	39.7 ± 8.2	39.9 ± 9.7	0.86
Lean mass (kg)	41.3 ± 6.7	40.8 ± 6.4	0.78
Body Fat (%)	48.8 ± 4.4	49.0 ± 5.1	0.45
Android fat (%)	54.3 ± 3.3	55.0 ± 4.0	0.49
Gynoid fat (%)	53.9 ± 5.3	54.5 ± 5.5	0.70
Android fat/Gynoid fat	1.0 ± 0.08	1.0 ± 0.08	0.77

Values are presented as mean ± standard deviation. PLA, placebo group; BARU, baru group.

**Table 2:** Dietary intake at baseline and after dietary intervention associated or not with baru almonds in overweight and obese women.

	PLA (*n* = 22)		BARU (*n* = 24)			Effect of Intervention		
	Baseline	Week 8	Baseline	Week 8	*P* ^a^	Time *P*	Diet *P*	Time × Diet *P*
Energy (kcal)	1575.66 ± 187.40	1604.60 ± 215.55	1713.48 ± 432.96	1742.71 ± 340.07	0.10	0.27	0.23	0.32
PTN (g)	78.15 ± 15.32	82.51 ± 12.75	84.34 ± 15.25	81.60 ±16.23	0.63	0.63	0.43	0.18
CHO (g)	212.12 ± 22.13	222.68 ± 18.79	218.04 ± 17.39	208.38 ± 15.78 *	0.69	<0.01	0.27	0.53
FAT (g)	45.61 ± 6.43	42.65 ± 7.82	55.99 ± 5.96	64.75 ± 6.23	0.57	0.89	0.03	0.35
SFA (g)	22.95 ± 6.95	26.03 ± 5.98	28.55 ± 8.75	30.57 ± 6.35	0.03	0.05	0.46	0.48
MUFA (g)	16.95 ± 3.23	16.19 ± 2.17	25.36 ± 4.59	30.34 ± 3.13 *	0.49	0.01	0.02	0.05
PUFA (g)	9.12 ± 0.13	12.03 ± 0.28	16.37 ± 0.54	22.71 ± 0.46 *	0.36	<0.01	<0.01	0.24
Fiber (g)	16.77 ± 5.18	16.04 ± 5.92	17.61 ± 7.93	20.53 ± 4.77	0.72	0.76	0.03	0.12

Values are presented as mean ± standard deviation. PLA, placebo group; BARU, baru group; PTN, protein; CHO, carbohydrates; SFA, saturated fatty acids; MUFA, monounsaturated fatty acids; PUFA, polyunsaturated fatty acids. * Difference between baseline and endpoint (*p* < 0.05). **^a^** Homoscedasticity test between groups at baseline.

**Table 3:** Biochemical, oxidative, and inflammatory parameters at baseline and after dietary intervention associated or not with baru almonds in overweight and obese women.

	PLA (*n* = 22)		BARU (*n* = 24)		Effect of Intervention			
	Baseline	Week 8	Baseline	Week 8	*P* ^a^	Time *P*	Diet *P*	Time × Diet *P*
Uric acid (mg/dL)	4.2 ± 1.5	4.1 ± 1.1	3.8 ± 0.8	3.6 ± 0.2	0.20	0.76	0.17	0.20
γGT (UI/L)	41.2 ± 24.8	32.4 ± 20.9	29.1 ± 19.2	25.0 ± 15.7 *	0.55	0.02	0.66	0.40
AST (UI/L)	23.4 ± 6.8	26.3 ± 10.3	21.7 ± 14.3	23.4 ± 17.9	0.95	0.01	0.50	0.61
ALT (UI/L)	25.0 ± 11.3	23.6 ± 9.8	23.1 ± 16.8	20.6 ± 11.2	0.75	0.46	0.28	0.99
Urea nitrogen (mg/dL)	29.6 ± 7.0	22.7 ± 5.7	26.9 ± 5.1	23.0 ± 5.6 *	0.15	<0.01	0.42	0.10
Creatinine (mg/dL)	0.80 ± 0.08	0.68 ± 0.13	0.78 ± 0.12	0.76 ± 0.14	0.42	0.73	0.48	0.08
CAT (U/mg)	7.9 ± 2.0	7.3 ± 1.9	8.2 ± 1.8	8.1 ± 2.7	0.90	0.49	0.43	0.49
GPx (U/mg)	0.42 ± 0.12	0.35 ± 0.10	0.32 ± 0.11	0.40 ± 0.1 *	0.33	0.30	0.76	<0.01
SOD (U/mg)	6.1 ± 1.6	5.7 ± 1.8	4.7 ± 1.6	6.0 ± 1.6 *	0.76	<0.01	0.33	0.84
MDA (nmol/mL)	0.97 ± 0.11	0.97 ± 0.14	0.99 ± 0.15	0.93 ± 0.25	0.20	0.39	0.34	0.45
IL-6 (pg/mL)	1.4 ± 0.44	0.82 ± 0.16	0.69 ± 0.09	0.68 ± 0.09	0.14	0.14	0.42	0.45
IL-10 (pg/mL)	3.8 ± 0.6	4.4 ± 0.7	4.0 ± 0.5	4.1 ± 0.5	0.53	0.89	0.42	0.68
ADP (µg/mL)	36.5 ± 5.2	40.2 ± 7.4	43.3 ± 8.1	43.5 ± 7.1	0.73	0.69	0.97	0.59
Insulin (U/mL)	12.8 ± 4.4	7.9 ± 0.9	7.7 ± 1.3	7.1 ± 0.9	0.22	0.49	0.28	0.06
TNF-α (pg/mL)	2.6 ± 0.26	2.0 ± 0.1	2.1 ± 0.1	2.2 ± 0.1	0.07	0.16	0.53	0.09

Values are presented as mean ± standard deviation. Abbreviations: PLA, placebo group; BARU, baru group; γGT, gama glutamil-transferase; AST, aspartate-amino-transferase; ALT, alanina-amino-transferase; MDA, malondialdehyde; CAT, catalase; GPx, glutathione peroxidase; SOD, superoxide dismutase; IL, interleukin; ADP, adiponectin; TNF-a, tumor necrosis factor-alpha. * Difference between baseline and endpoint (*p* < 0.05). ^a^ Homoscedasticity test between groups at baseline.

**Table 4:** Distribution of minerals in plasma samples at baseline and after dietary intervention associated or not with baru almonds in overweight and obese women.

	PLA (*n* = 22)		BARU (*n* = 24)		Effect of Intervention			
	Baseline	Week 8	Baseline	Week 8	*P* ^a^	Time *P*	Diet *P*	Time × Diet *P*
Zn (µg/L)	36.0 ± 5.6	35.6 ± 5.9	32.7 ± 5.2	32.6 ± 4.1	0.33	0.82	0.01	0.90
Ca (µg/L)	1.8 ± 2.6	1.8 ± 2.6	1.8 ± 2.6	1.8 ± 2.6	0.70	0.56	0.67	0.36
Fe (µg/L)	22.4 ± 7.8	19.2 ± 9.9	22.5 ± 2.2	18.6 ± 1.1	0.76	0.21	0.95	0.89
P (µg/L)	1.1 ± 4.2	1.1 ± 2.5	1.0 ± 1.6	1.1 ± 2.2	0.31	0.67	0.29	0.50
Mg (µg/L)	1.5 ± 1.8	1.5 ± 1.5	1.4 ± 2.0	1.4 ± 1.9	0.07	0.66	0.07	0.42
Co (µg/L)	0.58 ± 0.10	0.47 ± 0.12	0.47 ± 0.11	0.45 ± 0.14	0.02	<0.01	0.02	<0.01
Cu (µg/L)	15.2 ± 4.4	14.5 ± 3.6	12.8 ± 2.9	13.8 ± 0.03	0.03	0.76	0.11	0.03
Mn (µg/L)	24.4 ± 1.5	22.0 ± 2.7	35.8 ± 6.2	24.9 ± 3.1	0.45	0.85	0.37	0.51
Se (µg/L)	62.6 ± 23.9	66.3 ± 26.3	60.0 ± 18.8	58.9 ± 12.2	0.34	0.71	0.33	0.50
Sr (µg/L)	33.4 ± 10.3	32.3 ± 6.8	30.6 ± 8.2	29.6 ± 6.6	0.34	0.38	0.19	0.99

Values are presented as mean ± standard deviation. Abbreviations: PLA, placebo group; BARU, baru group; Zn, zinc; Ca, calcium; Fe, iron; P, phosphor; Mg, magnesium; Co, cobalt; Cu, copper; Mn, manganese; Se, selenium; Sr, strontium. **^a^** Homoscedasticity test between groups at baseline.
